# Sex linked behavioral and hippocampal transcriptomic changes in mice with cell-type specific *Egr1* loss

**DOI:** 10.3389/fnins.2023.1240209

**Published:** 2023-10-19

**Authors:** Cody Swilley, Yu Lin, Yuze Zheng, Xiguang Xu, Min Liu, Timothy Jarome, Georgia E. Hodes, Hehuang Xie

**Affiliations:** ^1^Epigenomics and Computational Biology Lab, Fralin Life Sciences Institute, Virginia Tech, Blacksburg, VA, United States; ^2^Department of Biomedical Sciences and Pathobiology, Virginia-Maryland College of Veterinary Medicine, Virginia Tech, Blacksburg, VA, United States; ^3^Genetics, Bioinformatics and Computational Biology Program, Virginia Tech, Blacksburg, VA, United States; ^4^School of Animal Sciences, Virginia Tech, Blacksburg, VA, United States; ^5^School of Neuroscience, Virginia Tech, Blacksburg, VA, United States

**Keywords:** sex difference, *Egr1*, nestin, RNA-seq, hippocampus, behavior

## Abstract

The transcription factor EGR1 is instrumental in numerous neurological processes, encompassing learning and memory as well as the reaction to stress. *Egr1* complete knockout mice demonstrate decreased depressive or anxiety-like behavior and impaired performance in spatial learning and memory. Nevertheless, the specific functions of *Egr1* in distinct cell types have been largely underexplored. In this study, we cataloged the behavioral and transcriptomic character of Nestin-Cre mediated *Egr1* conditional knockout (Egr1cKO) mice together with their controls. Although the conditional knockout did not change nociceptive or anxiety responses, it triggered changes in female exploratory activity during anxiety testing. Hippocampus-dependent spatial learning in the object location task was unaffected, but female Egr1cKO mice did exhibit poorer retention during testing on a contextual fear conditioning task compared to males. RNA-seq data analyses revealed that the presence of the floxed *Egr1* cassette or Nestin-Cre driver alone exerts a subtle influence on hippocampal gene expression. The sex-related differences were amplified in Nestin-Cre mediated *Egr1* conditional knockout mice and female mice are more sensitive to the loss of *Egr1* gene. Differentially expressed genes resulted from the loss of *Egr1* in neuronal cell lineage were significantly associated with the regulation of Wnt signaling pathway, extracellular matrix, and axon guidance. Altogether, our results demonstrate that Nestin-Cre and the loss of *Egr1* in neuronal cell lineage have distinct impacts on hippocampal gene expression in a sex-specific manner.

## Introduction

The early growth response factor 1 (*Egr1*) is an early response gene that can be rapidly and transiently induced in response to various environmental stimuli (Gashler and Sukhatme, 1995; Li et al., 2005; Veyrac et al., 2014). As a transcription factor, the EGR1 protein directly binds to genomic DNA containing a motif with CpG dinucleotides and serves as a critical epigenetic regulator for downstream gene activation (Sun et al., 2019). Through the regulation of gene expression, *Egr1* plays an essential role in learning and memory processes (Gallo et al., 2018). Compared to wild-type mice, *Egr1* complete knockout mice showed deficits in long-term potentiation (LTP) and long-term depression (LTD) in the hippocampus. The performance of *Egr1* complete knockout mice was impaired in various behavioral tests including spatial memory and object recognition memory (Jones et al., 2001; Bozon et al., 2003). *Egr1* is also involved in the stress response and has been implicated in various stress-related disorders, including depression and anxiety. For instance, decreased depressive-like behavior was observed in rats with local infusion of antisense oligodeoxynucleotide to specifically knockdown *Egr1* in the dorsal hippocampus (Katche et al., 2012). Additionally, initial learning or retrieval of an auditory or contextual fear memory induced increases in *Egr1* expression in the amygdala or hippocampus, respectively, and the knockdown of *Egr1* impaired long-term memory for both tasks (Lee et al., 2004; Maddox et al., 2011). A previous study has investigated the effects of *Egr1* loss on hippocampal genome-wide gene expression using cDNA microarray (Han et al., 2014). A total of 368 differentially expressed genes (DEGs) were identified in the hippocampi from *Egr1* complete knockout mice exposed to different phases of a fear conditioning paradigm. Despite these previous studies performed with complete knockout mice or regional *Egr1* knockdown with antisense oligodeoxynucleotide, little is known about the changes in behavior or gene expression profile associated with the cell type-specific loss of *Egr1*.

Male and female mice are reported to have different behavioral responses to external stimuli (Yang and Shah, 2016). For instance, stress reactivity and depression prevalence are different between the sexes, with women of reproductive age being more susceptible to depression/stress. Decades of studies have broadened our comprehension of how EGR1 regulates hippocampal function, particularly in relation to sex differences. An early report has shown that the expression levels of *Egr1* and other immediate early genes in the hippocampus were differentially regulated during the formation of the intromission mnemonic, which is a learned association between sexual behavior and reward (Yang et al., 2007). Normal aggressive behaviors and social recognition memory are dependent on the proper functioning of vasopressin 1b receptor (Avpr1b) in the CA2 region of the hippocampus. In Avpr1b knockout mice, the intruder-evoked *Egr1* expression was observed in male but not in female mice (Witchey et al., 2016). Sex differences in corticosterone receptors in the hippocampus has been reported (Lin et al., 2011). Only in female mice with corticosteroid-binding globulin knockout, the mRNA expression of *Egr1* was reduced in the hippocampus after the stress induced by the force swim test (Minni et al., 2014). However, the influence of *Egr1* loss on sex-linked behavior and hippocampal gene expression profile remains unexplored.

The Nestin-Cre transgenic mouse is one of the most frequently used mouse models to study gene functions in the central nervous system. In this study, we used the Nestin-Cre driver to obtain *Egr1* conditional knockout (Egr1cKO) mice with the neuronal-specific loss expression of the *Egr1* gene. We examined the behavior changes and evaluated the sex-related differential gene expression in the hippocampi of Egr1cKO mice along with Nestin-Cre controls.

## Materials and methods

### Animals

All animal experiments were performed according to the Institutional Animal Care and Use Committee guidelines at Virginia Tech (Blacksburg, VA, USA). The *Egr1* conditional knockout mouse strain (Egr1_tm1a_A04, C57BL/6 N-Egr1/Tcp; MGI:5766027) was purchased from the Centre for Phenogenomics, Canada. The Nestin-Cre (B6.Cg-Tg (Nes-Cre)1Kln/J; Jackson Lab, #003771) was a kind gift from Dr. Michael Fox’s lab. To create the Egr1cKO mice, a breeding scheme was implemented to obtain the desired Nestin-Cre driving Egr1cKO (Swilley et al., 2023). The mice were maintained and bred in a 12-h light/dark cycle between 7:00 am and 7:00 pm, under standard pathogen-free conditions. Teklad Global 18% Protein Rodent Diet was provided with free food and water access.

### Genotyping PCR

Mice were genotyped at 3 weeks of age during the time of weaning. A small distal portion of the tail was removed and incubated at 55°C overnight in DirectPCR (Tail) (VIAGEN, cat# 101-T) solution along with Proteinase K (ThermoFisher, AM2546). The next day the sample was incubated at 80°C for 1 h to deactivate the proteinase K before setting up PCR sampling. Genotyping PCR reactions were performed according to the Jackson Laboratory’s protocol for Nestin-Cre and the Centre for Phenogenomics’ protocol for *Egr1*-loxp as described in our previous study (Swilley et al., 2023).

### Western blotting

Total proteins were extracted from tissues using lysis buffer, separated in the 10% SDS-PAGE gel, and transferred to PVDF membranes. After blocking with 5% skimmed milk for 1 h, and then the membrane was incubated with the primary antibodies at 4°C overnight. The primary antibodies against EGR1 (CST, cat# 41542, 1:1000 dilution) and GAPDH (CST, cat# 2118, 1:2000 dilution) were used. The next day, the membrane was incubated with specific secondary antibodies at 25°C for 1 h. After three washes with PBST buffer, the membrane was incubated with Super Signal West Pico PLUS Chemiluminescent Substrate (Thermo Fisher, cat# 34580), and visualized using the Bio Rad ChemiDoc imaging system. GAPDH was included as an internal control.

### Immunohistochemistry (IHC)

Egr1cKO mice and controls were rapidly and deeply anesthetized with isoflurane (Vet One, cat# 502017) and perfused transcardially with 10% formalin into the left ventricle. The right ventricle was opened to allow for exsanguination. After cardiac arrest was confirmed, the mice went through cervical dislocation. The brain was exposed with maximum bone removed to allow for further tissue fixation in 10% formalin overnight. Once fixed, tissues were sent to the Virginia-Maryland College of Veterinary Medicine pathology laboratory for embedment. Coronal slices were taken of the brain near the hippocampus in the wax block and provided back to the pathology laboratory. The coronal brain sections went through an automotive system with a Ventana Discovery Ultra machine (DAB Detection kit Cat#: 760–159) and secondary antibody, Omap anti-Rb HRP (Roche, Cat#: 760–4,311). The brain section was stained with anti-EGR1 rabbit antibody (CST, cat# 41542). Images were acquired using a MoticEasy Scan Pro 6, slide scanner.

### General procedure for animal behavior tests

All animals were habituated to the testing room 30 min prior to each test. All behavior testing started at 2:00 pm each day regardless of test or cohort. The testing cohorts were allowed 24 h in between different behavioral assays before going on to the next test. Most behavioral tests were performed in red light, to not increase anxiety levels from the environment; normal lighting conditions were used for the two sensory tests with heat for protection of the administrator of the test and the animals. To minimize other factors such as sex impeding on results of different cohorts all male animals went through behavior testing prior to any female testing. All testing arenas or devices were cleaned with 75% alcohol before and after each test was performed.

### Open field

Noldus information Technology Inc. behavioral system Ethos XT 17 was used to track behavior in the open field. Predefined Open field arena from the Ethos XT 17 software was used as a template and then modified for specific parameters set for the open field. A square box approximately 50 cm x 50 cm x 50 cm was used for the open field arena. The mouse was placed into the center of the box and allowed to explore for 10 min; after the time was complete, the mouse was removed and returned to its home cage.

### Elevated plus maze

An elevated plus maze testing apparatus from Clever Systems Inc. was used for testing (CSI-MZ-EP-M). It has two open arms with a combination measurement of (CSI-MZ-M: Open Arm 7 cm width, 69 cm length, and 0 cm height) and two closed arms with a combination measurement of (CSI-MZ-M: Open Arm 7 cm width, 30 cm length, and 15 cm height). Tracking was performed using Noldus Ethovision (Ethos XT 17). The mouse was placed into the open arm and allowed to explore for 10 min. The mouse was then removed and returned to its home cage. The elevated plus maze apparatus was cleaned with 75% alcohol between each test.

### Hot plate

The hotplate test was conducted using the Hotplate Analgesia meter from Columbus Instruments (serial #210028). The tests began when the system reached 55°C. A timer was started when each mouse was placed inside the apparatus one by one and monitored for signs of thermal discomfort such as flicking of the tail and limbs, or combination of signs. Some mice did displace other signs such as immediate launching to the sides of the walls around the meter. Once signs were documented timing stopped and the mouse was removed to prevent any thermal injury. If there was no response within 15 s, the test was terminated, and the mouse was removed from the apparatus to prevent thermal injury.

### Tail flick

Maze Engineers tail flick device was used to test each individual mouse. Mice were restrained in an A refrainer device (Maze Engineers). The tail flick device recorded the timing of how long the mouse kept the tail over the light source. All mice were removed after 15 s if no flick was present, to prevent any possible thermal injury.

### Novel spatial object

Noldus Information Technology Inc. behavioral system Ethos XT 17 was used for tracking. A square box approximately 50 cm x 50 cm x 50 cm was used for the arena and one plastic square (5 cm x 5 cm x 5 cm) and one tin can (5 cm x 5 cm x 10 cm) was used as the objects during testing. The Novel object testing occurred over 6 days for 10 min per day. Day 0 was used for habituation to the for the arena with no objects. During days 1–4 objects were placed in the arena and the mouse was allowed to explore for 10 min. Testing was performed on Day 5 when one of the objects was moved to a new location. The object moved was counterbalanced across trials.

### Contextual fear conditioning

Freeze Frame (Version 4.201) from Actimetrics software was used to score freezing behavior. The software worked in conjunction with Coulbourn habitest isolation cubicle (model # H10-24TA) and precision animal shocker (Serial #802313, Catalog # H13-15) to administer the contextual fear conditioning protocol. The mice went through novel spatial object testing, prior to contextual fear conditioning, which provided several days of handling and transportation. Each mouse was transported one by one to the testing room on day 0 which was the contextual fear conditioning training day. During this, two-foot shock presentations (1 s, 0.4 mA, 60 s inter-trial interval) were given after a 3 min baseline period. Animals were removed after being in the chamber for 5 min. The chamber was cleaned prior to each test experiment and wiped with 75% ethanol. The next day each mouse was placed back into the chamber without any shock presentations and monitored for freezing behavior across a 5 min period.

### Statistical analysis

All behavioral data was analyzed with a two-way factor ANOVA (genotype x sex), with an alpha set to 0.05. Tukey’s HSD was used for *post-hoc* analysis.

### RNA extraction, RNA-seq library construction, and data analysis

RNA was extracted from the hippocampus using the RNAeasy Mini Kit (Qiagen, Cat# 74104). Homogenized samples were subjected to the protocol provided by the manufacturer. The RNA concentrated on the silica membrane was eluted with RNase-free molecular biology-grade water. 150 μg total RNA collected from each tissue sample was shipped to Novogene Corporation Inc. for RNA-seq library construction (Illumina). The libraries were sequenced on Novaseq 6,000 platform with 150 bp paired-end mode (Illumina). Trim Galore (version [0.6.5]) was used to filter short reads, low quality reads and trim adapter sequences from raw reads. Clean reads were mapped to the mm10 genome, and expression was quantified using STAR[(version2.7.3a)] (Dobin et al., 2013). The raw counts were employed to pair wisely identify differentially expressed genes by R package DESeq2 (Love et al., 2014). Benjamini-Hochberg method is used to adjust the value of *p* for DEG. Gene with expression change greater than or equal to 1.2-fold and adjusted value of *p* less than or equal to 0.05 were considered significant. R package glmmSeq was used to perform mix-model analysis which took floxP and Cre as two mixed-effect factors and sex as an additional factor, while using the sample ID (unique to each sample) as random effect variable for glmmSeq analysis (Rivellese et al., 2022).

### Go analysis

Gene Ontology (GO) analysis was performed and visualized using R package clusterProfiler (v4.4.4) (Wu et al., 2021). Default parameters were used for the enrichment analysis for biological process (BP). The resulting GO terms and the corresponding values of *p* were then processed using R package rrvgo to reduce GO term redundancy (Sayols, 2023). Benjamini-Hochberg method was used to adjust the value of *p* for GO terms, and the cutoff was set as the adjusted value of *p* at 0.05.

## Results

### Behavioral changes observed in Nestin-Cre and Egr1cKO mice

**Figure 1:**
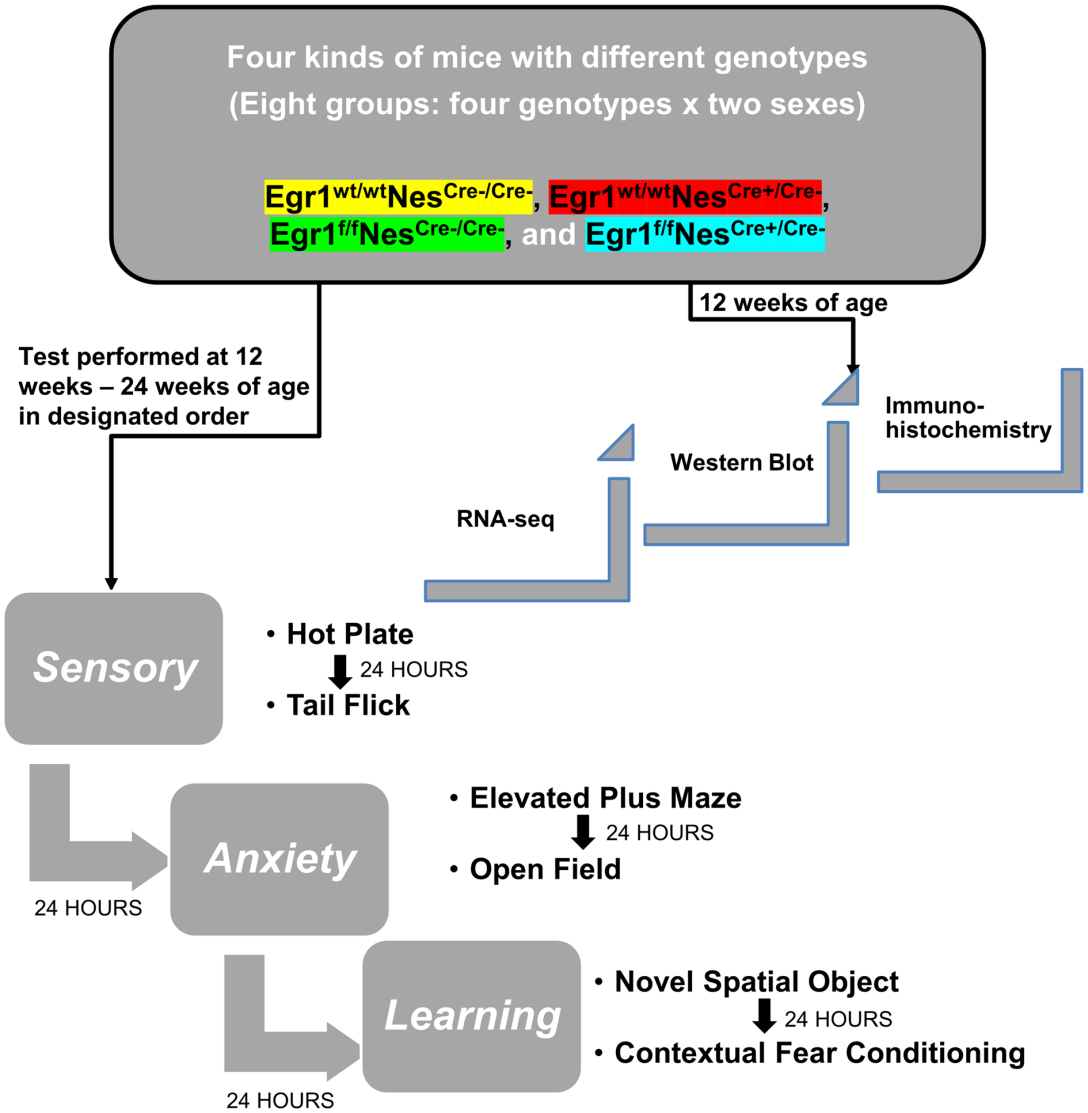
Diagram of experimental design and behavioral tests. Experimental design for gene expression and behavioral test scheme to explore the role and mechanism of Egr1 in Nestin-Cre driving conditional knockout mouse model.

Although previous research has extensively studied the behavioral changes of mice with *Egr1* complete knockout, no data has been collected on the effects associated with *Egr1* loss in neuronal lineage cells. In this study, we used a Sex X Genotype design. Genotype was subdivided across 4 groups (Egr1^f^Nes^cre+^, Egr1^f^Nes^cre-^, Egr1^wt^Nes^cre+^, and Egr1^wt^Nes^cre-^) and tested by sex (male/female) resulting in a total of 8 groups. As shown in Figure 1, we conducted a behavioral test battery to evaluate pain sensitivity, anxiety-like behavior, spatial, and aversive learning/memory (For each behavioral test, *n* = 10 per sex/group for a total of 80 mice).

**Figure 2:**
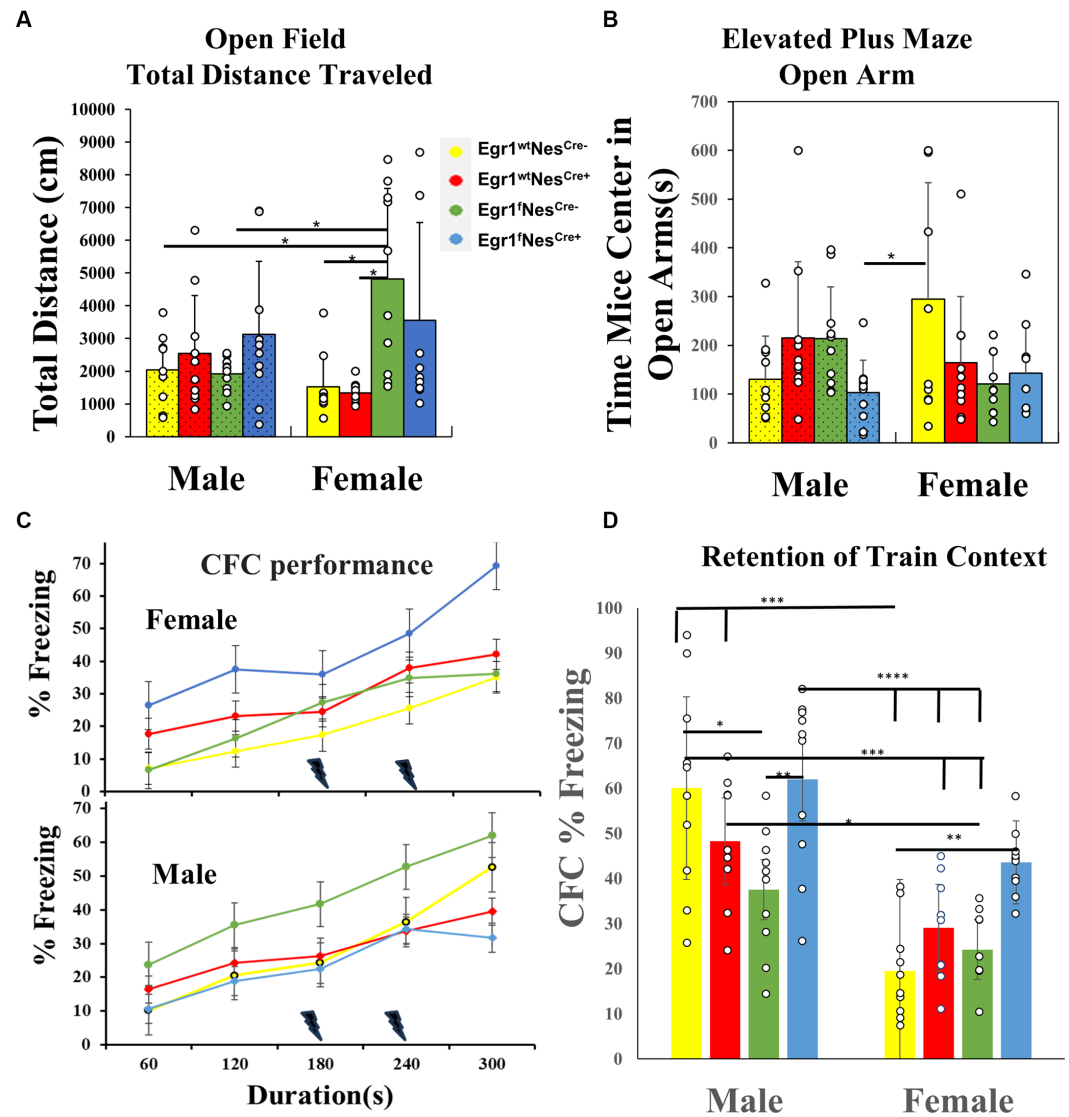
Behavioral test results for male and female mice of four genotypes. **(A)** Open Field Total distance traveled (cm) bar chart from males and females, with significance bars and * shown for sex and genotype. **(B)** Elevated Plus Maze Open Arms bar chart of time spent in open arms(s) for males and females. Significance is marked with a bar and * between groups. **(C)** Contextual Fear Conditioning line chart on training day for freezing (%) of females (upper panel) and males (lower panel) only. Each point represented the mean during that minute of training in the CFC task. A lighting denotes the times that a shock was administered during the test. **(D)** Contextual Fear Conditioning freezing response (%) during the testing session (first day after training) for both males and females. Bars represent means for genotypes over the entire freezing session. Significance above groups between sex and genotype along with * to denote level of significance. The data for each time point in each test was derived from 10 individuals per group and presented as means ± SEM. Each bar is separated by sex and then genotype within sex and represented by color as well as males have textured bars. * denotes *p*-value that is significant, with different amounts of stars equating to different level of *p* values, *p < 0.05, **p < 0.005, *** *p* < 0.001 and ****p < 0.0001.

The tail flick test measures the sensitivity of A-delta fibers with a mild and short-lasting nociceptive stimulus, while the hot plate test measures the sensitivity of C fibers with a more intense and enduring nociceptive stimulus (Afify et al., 2017). For the hot plate test and tail flick (Supplementary Figures S1A,B), no difference occurred across genotype and/or sex indicating all pathways are still physiologically intact and there were no changes in pain responses. The open field test measured exploratory anxiety-like and locomotor behavior. None of the eight groups showed any difference in time spent in the center of the open field (Supplementary Figure S1C); however, the total distance traveled during the open field test did have a significant interaction between genotype and sex [*F* (3, 70) = 5.15, *p* < 0.05]. *Post hoc* testing with Tukey’s revealed the female group Egr1^f^Nes^Cre-^ (6525.99 ± 875.2 cm) had a ~ 4–5 fold higher distance traveled in open field than female groups Egr1^wt^Nes^Cre-^ (1524.996 ± 292.97 cm) and Egr1^wt^Nes^Cre+^ (1331.541 ± 99.69 cm). Female Egr1^f^Nes^Cre-^ mice were also significantly different from (*p* < 0.05) male Egr1^f^Nes^Cre-^ mice (1915.66 ± 165.9 cm) and Egr1^wt^Nes^Cre –^ mice (2034.922 ± 328.72 cm) with an ~3-fold difference in distance traveled (Figure 2A). As an additional measure of exploratory anxiety, we used the elevated-plus maze. This test measures a conflict between exploratory risks in the open arm vs. the safety signals of the closed arms. There was a significant interaction between genotype and sex with *F* (3, 72) = 3.81, *p* < 0.05. *Post hoc* analysis with Tukey’s found that female Egr1^wt^Nes^Cre –^ mice (294.82 ± 75.58 s) spent 2.8-fold more time in the open arms compared to the male knockout group Egr1^f^Nes^Cre+^ (103.39 ± 20.98 s). We did not identify any within sex effects of knockout (Figure 2B). Together, these data identify that neuron-selective deletion of *Egr1* within sex did not alter anxiety associated behavior, although it did alter the relationship between activity levels and anxiety between the sexes.

To determine perceptual learning and recognition memory, we performed the novel object spatial recognition tests which revealed no significant effect regardless of sex or genotype and demonstrated that spatial awareness and object recognition are not altered (Supplementary Figure S1D). To examine the process of learning to associate aversive stimuli with environmental information, we conducted contextual fear conditioning. During training, genotype affected the amount of time animals froze across shock exposures in a sex-specific manner with significant interaction *F* (3, 32) = 4.194, *p* < 0.05. *Post hoc* analysis did not indicate a specific relationship in the groups. The Egr1^f^Nes^Cre+^ male group was the only group to have a decrease in freezing responses after the second shock (Figure 2C), though all groups had increased freezing across training. To test memory retention, freezing behavior in the training context in the absence of shock was used. There was a significant interaction between sex and genotype during testing *F* (3, 71) = 3.557, *p* < 0.05, as well as a main effect of sex *F* (1, 71) = 50.37, *p* < 0.001, and genotype *F* (3, 71) = 8.023, *p* < 0.0001 (Figure 2D). *Post hoc* analysis with Tukey’s identified the following differences within sex. Egr1^f^Nes^Cre+^ males had a higher percentage of freezing during the test than the Egr1^f^Nes^Cre-^ males (*p* < 0.01) Within females, Egr1^f^Nes^Cre+^ also froze more during test than Egr1^wt^Nes^Cre-^ (*p* < 0.01). Overall females froze less than males during the testing session (*p* < 0.05).

### Sex differences in gene expression amplified in Egr1cKO mice

In addition to cataloging behavioral effects of neuron specific Egr1cKO and Nestin-Cre, we examined their hippocampal gene expression patterns given the previously published reports of whole-body KO resulting in learning impairment. To accomplish this, RNA-seq was conducted in triplicates using adult hippocampal tissues obtained from the eight groups of mice. Upon analyzing the transcriptomic data using Principal Component Analysis (PCA), we found that the twenty-four samples could be separated into two groups based on their gene expression profiles. The female mice were positioned on the left upper side of the graph while the males were positioned on the right (Supplementary Figure S2A). Pearson’s correlation of gene expression across samples demonstrated sample variation within groups, but neither sex nor genotype is a good clustering parameter (Supplementary Figure S2B). We observed a high degree of correlation among the samples, with a mean correlation coefficient of 0.988, and the lowest correlation observed between samples was 0.953. These results indicate that hippocampal gene expression shows a high level of consistency both within and across groups, irrespective of their differing genotypes. It’s worth mentioning that, for 22 out of the 24 mice included in this study, we have conducted transcriptomic analysis on pituitary tissues in our previous study (Swilley et al., 2023). A remarkable contrast in pituitary gene expression was evident, with an average correlation of 0.797 and the lowest correlation recorded at 0.307.

**Figure 3:**
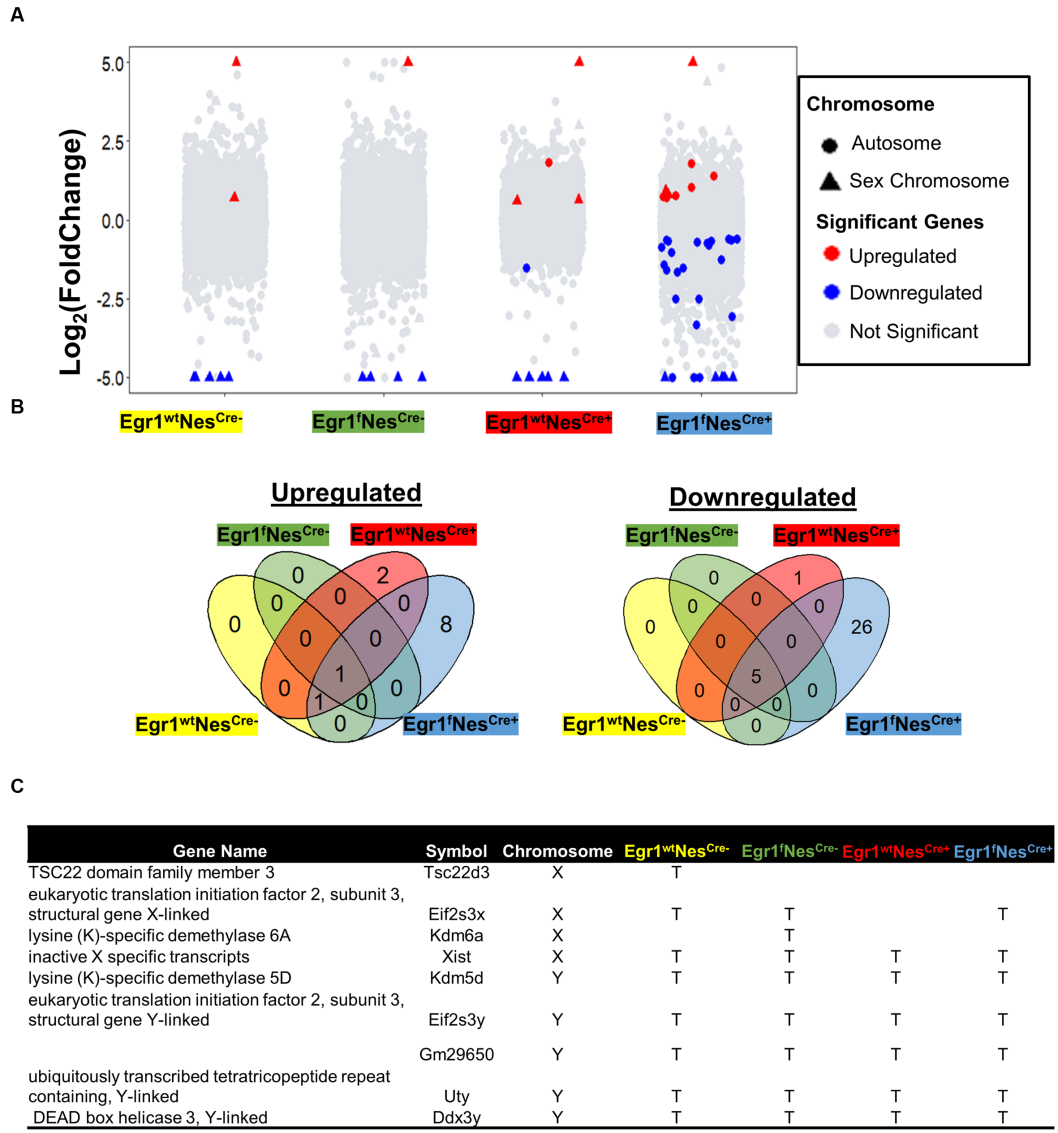
Gene expression changes associated with sex difference. **(A)** Volcano plots for pairwise analyses between two sexes for four genotypes. DEGs were defined as genes with FC ≥ 1.2 and adjusted *p* value ≤ 0.05. Blue dots indicate DEGs down-regulated in males. Red dots indicate DEGs up-regulated in males; Grey dots indicate no DEGs. **(B)** Venn diagram showing the overlapped DEGs identified in pairwise comparisons. Corresponding genotypes were shaded with represented color (blue for Egr1^f^Nes^cre+^, green for Egr1^f^Nes^cre-^, red for Egr1^wt^Nes^cre+^and yellow for Egr1^wt^Nes^cre-^). **(C)** A list of DEGs on sex chromosomes.

We next performed pairwise comparisons for four genotypes separately to identify genes associated with sex-specific hippocampal expression. For wild-type mice (Egr1^wt^Nes^Cre-^), a total of 7 genes were identified with 5 downregulated and 2 up-regulated in females (Figure 3A). The numbers of DEGs involving in sex-related difference were 6 in Egr1^f^Nes^Cre-^, 10 in Egr1^wt^Nes^Cre+^, but increased to 41 in Egr1^f^Nes^Cre+^ mice. The majority of DEGs identified between the two sexes were Egr1cKO-specific and ten out of total 44 DEGs identified were derived from sex chromosomes (Figure 3B). Only 1 gene up-regulated and 5 genes down-regulated in females were found to be shared by all four genotypes. These shared genes are all from sex chromosomes, such as Kdm5d on the Y chromosome and Xist on the X chromosome (Figure 3C).

To further examine whether the small sample size (*n* = 3) resulted in such a few DEGs detected, we divided twenty-four mice into two groups based on sex to perform pair-wise comparison. The increasing group size (*n* = 12) did not yield much more DEGs with only 18 DEGs determined (Supplementary Figure S3A). Although the majority of them show consistent expression profiles within sex groups (Supplementary Figure S3B), significant variation within female group was observed for two genes encoding for transporters, the sodium bicarbonate cotransporter gene (SLC4A5) and transthyretin (TTR) gene which transports vitamin A and thyroxine. Therefore, these two genes should be excluded from the sex-linked DEG list. Altogether, we concluded that only a limited number of genes are differentially expressed between male and female mouse hippocampi. Such a small sex difference is primarily driven by genes on sex chromosomes. Nonetheless, the absence of the *Egr1* gene amplifies the sex-related differences in the hippocampus, affecting 21 genes spread across autosomes, with half of them having unknown functions. Interestingly, some genes such as *Fosl2*, *Neat1*, and *Col5a3* have been reported under the regulation of estrogen (Klinge, 2020; Göcz et al., 2022; Sharma et al., 2023).

### Aberrant gene expression associated with nestin-Cre, Floxp, and Egr1cKO

To understand the effect of the Nestin-Cre driver and Floxp on hippocampal expression, we first kept male and female RNA-seq data separately to perform pairwise comparisons (Supplementary Figure S4). Interestingly, no gene was found to be differentially expressed in male groups and only 6 DEGs were identified in female groups with significant Fold Change (FC) and adjusted *p*-values below 0.05. Compared with Egr1^wt^Nes^Cre-^, four genes were upregulated in female Egr1^f^Nes^Cre-^, including MFSD2 Lysolipid Transporter A (Mfsd2a, FC = 1.67, *p* = 0.044), Perilipin 4 (Plin4, FC = 3.83, *p* = 0.042), Solute Carrier Family 2 Member 1(Slc2a1, FC = 1.63, *p* = 0.045), and Xanthine Dehydrogenase (Xdh, FC = 2.42, *p* = 0.042). Compared with Egr1^wt^Nes^Cre-^, Heat shock protein beta-8 (Hspb8, FC = 1.57, *p* = 0.045) was upregulated and TCDD Inducible Poly (ADP-Ribose) Polymerase (Tiparp, FC = 1.83, *p* = 0.003) was downregulated in female Egr1^wt^Nes^Cre+^. Worthy of mentioning, Hspb8 and its α-crystallin domain might act as pleiotropic pro-survival factor in the adult hippocampus (Ramírez-Rodríguez et al., 2013). TIPARP is an enzyme involved in molecular cascades regulating structural changes in synaptic connections and correct distribution and number of GABAergic neurons (Grimaldi et al., 2019). Very few DGEs identified with *p*-values on the edge of thresholds suggest that the presence of Floxp or Nestin-Cre driver alone may only have subtle impact on female hippocampal functions.

**Figure 4:**
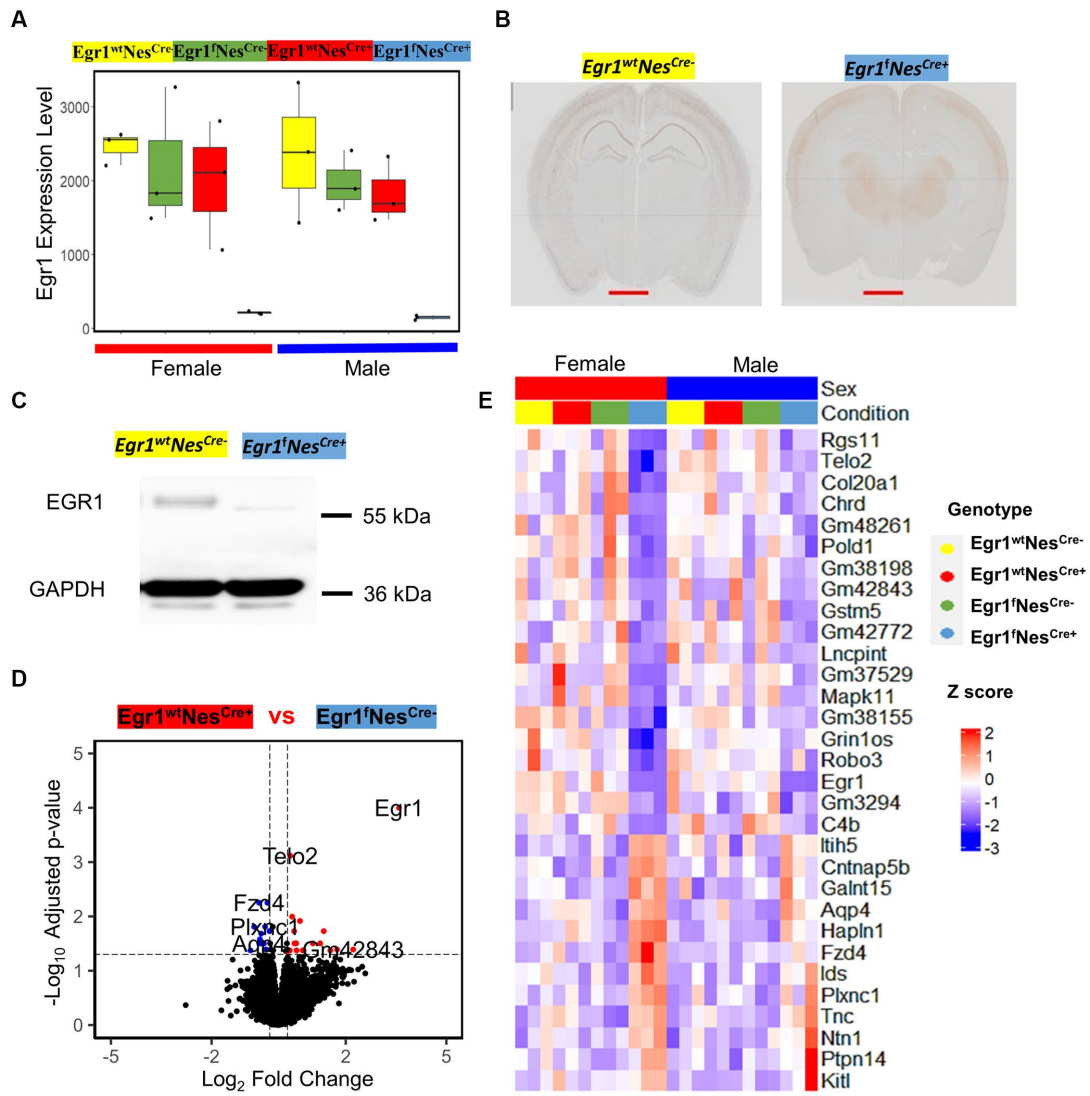
The expression of *Egr1* mRNA and EGR1 protein in the hippocampus of Egr1cKO mice and differential expressed genes associated with Egr1cKO identified by pairwise comparison. **(A)** Box plots for *Egr1* expression levels in mice of four kinds of genotypes determined by RNAseq. **(B)** Immunohistochemistry staining of EGR1 protein expression in mouse brain (coronal view, scale bar = 2 mm). **(C)** Western blot of EGR1 protein expression in mouse hippocampus, GAPDH serves as internal control. **(D)** Volcano plot for pairwise analysis between mouse hippocampi with and without Egr1cKO. DEGs were defined as genes with FC ≥ 1.2 and adjusted *p* value ≤ 0.05. Blue dots indicate DEGs down-regulated in *Egr1* wildtype controls. Red dots indicate DEGs up-regulated in *Egr1* wildtype controls; Black dots indicate no DEGs. **(E)** Heatmap of gene expression profiles for DEGs identified.

We next focused on the transcriptomic analysis of Egr1cKO mice and observed that the expression of *Egr1* gene is highly expressed in wild type mice but abolished in the hippocampi of both female and male mice (Figure 4A). To validate the changes of *Egr1* expression at protein level, immunohistochemistry using anti-EGR1 antibody was performed on brain sections obtained from Egr1^f^Nes^cre+^ and Egr1^wt^ Nes^cre-^ adult mice (Figure 4B). EGR1 proteins are highly expressed in the hippocampus in Egr1^wt^ Nes^cre-^ mice but substantially diminished in that of Egr1^f^Nes^cre+^ mice. Further western blot also demonstrated that the level of EGR1 proteins was significantly reduced in the hippocampus of Egr1cKO mice (Figure 4C). Considering the similar transcriptomic profiles observed in both sexes of wild type mice, we divided the 24 mice into two groups: 18 with high *Egr1* expression and 6 with the loss of *Egr1* in cell lineages expressing Nestin gene. Pair-wise comparison determined 12 genes upregulated and 19 genes downregulated in Egr1cKO hippocampus (Figure 4D). Heatmap of the expression profiles of these genes indicated that female show more striking and consistent changes than male counterparts (Figure 4E).

**Figure 5:**
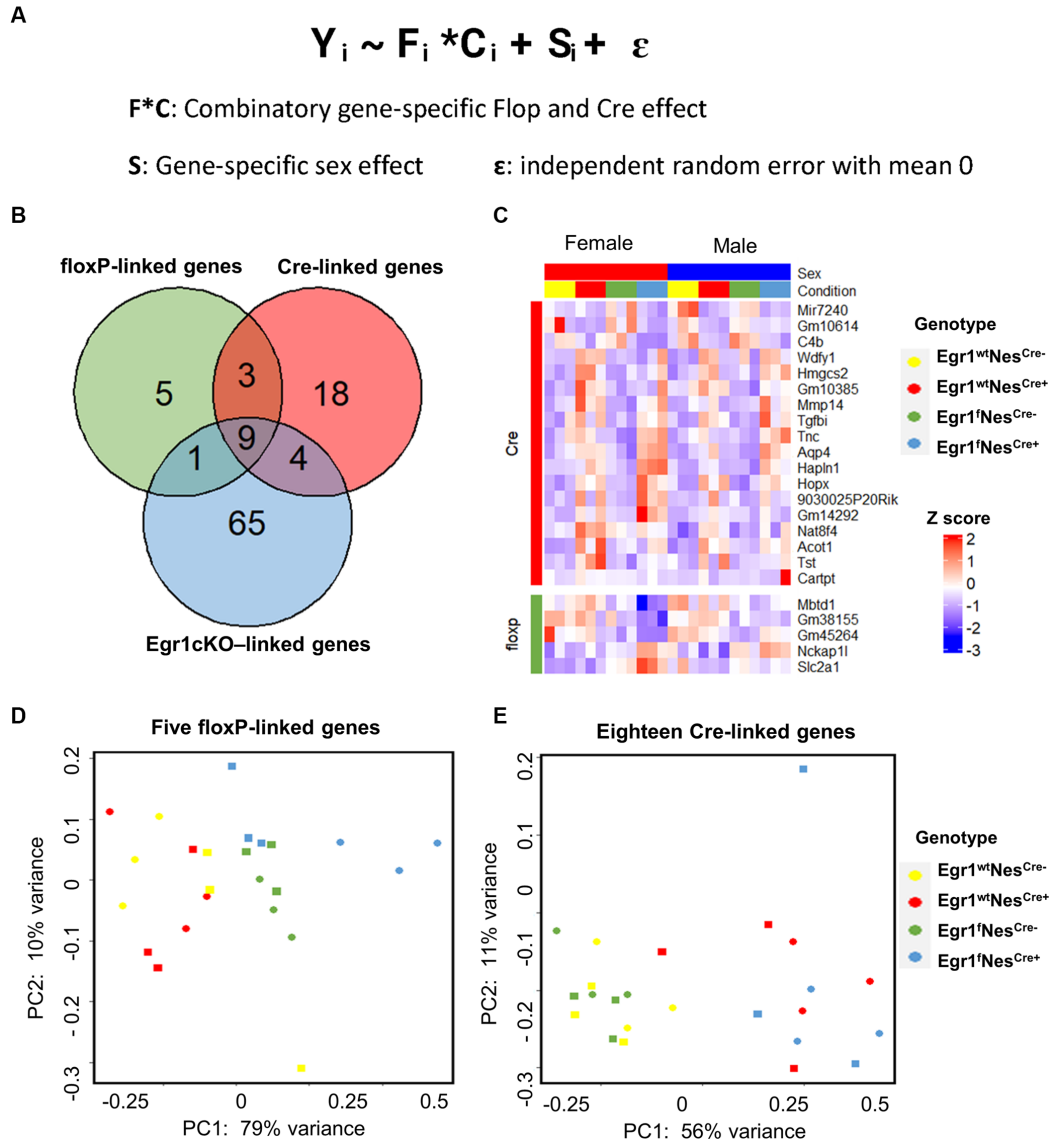
Differential gene expression analysis using a mixed-effects model. **(A)** Formula of mixed-effects model. **(B)** Venn diagram showing the overlapped DEGs identified. **(C)** Heatmap of gene expression profiles for DEGs identified to be linked with floxP or Nestin-Cre. **(D,E)** PCA analyses for five floxP-linked **(D)** and 18 Nestin-Cre-linked genes **(E)**.

In this study, four factors (sex, flop, Nestin-Cre, and the loss of *Egr1* expression) may contribute to hippocampal gene expression changes. Since pairwise comparison is not sufficient to scrutinize the influence of these four factors simultaneously, we adopted a mixed effect model to analyze the hippocampal gene expression profiles from the mice of all four genotypes (Figure 5A). In this mixed model, the combination of Floxp and Nestin-Cre would lead to the loss of *Egr1*. Interestingly, nine genes including *Egr1* were shown to be under the influence of floxP, Nestin-Cre, and Egr1cKO (Figure 5B). Five genes were determined to be specifically associated with floxP genotype and 18 genes were linked to Nestin-Cre (Figures 5B,C). Further PCA analyses using the expression patterns of these genotype-linked genes are sufficient to separate the mice into groups corresponding to their genotypes (Figures 5D,E). The PC1 variances, the amount of variability in a data set that can be attributed to first principal component, are 79% for floxP-linked genes and 56% for 18 Nestin-Cre linked genes. Therefore, pairwise comparisons are straight forward to identify expression differences between two given groups but a mixed effect model is powerful to reveal underlying changes associated with multiple factors in a complicated experimental design.

**Figure 6:**
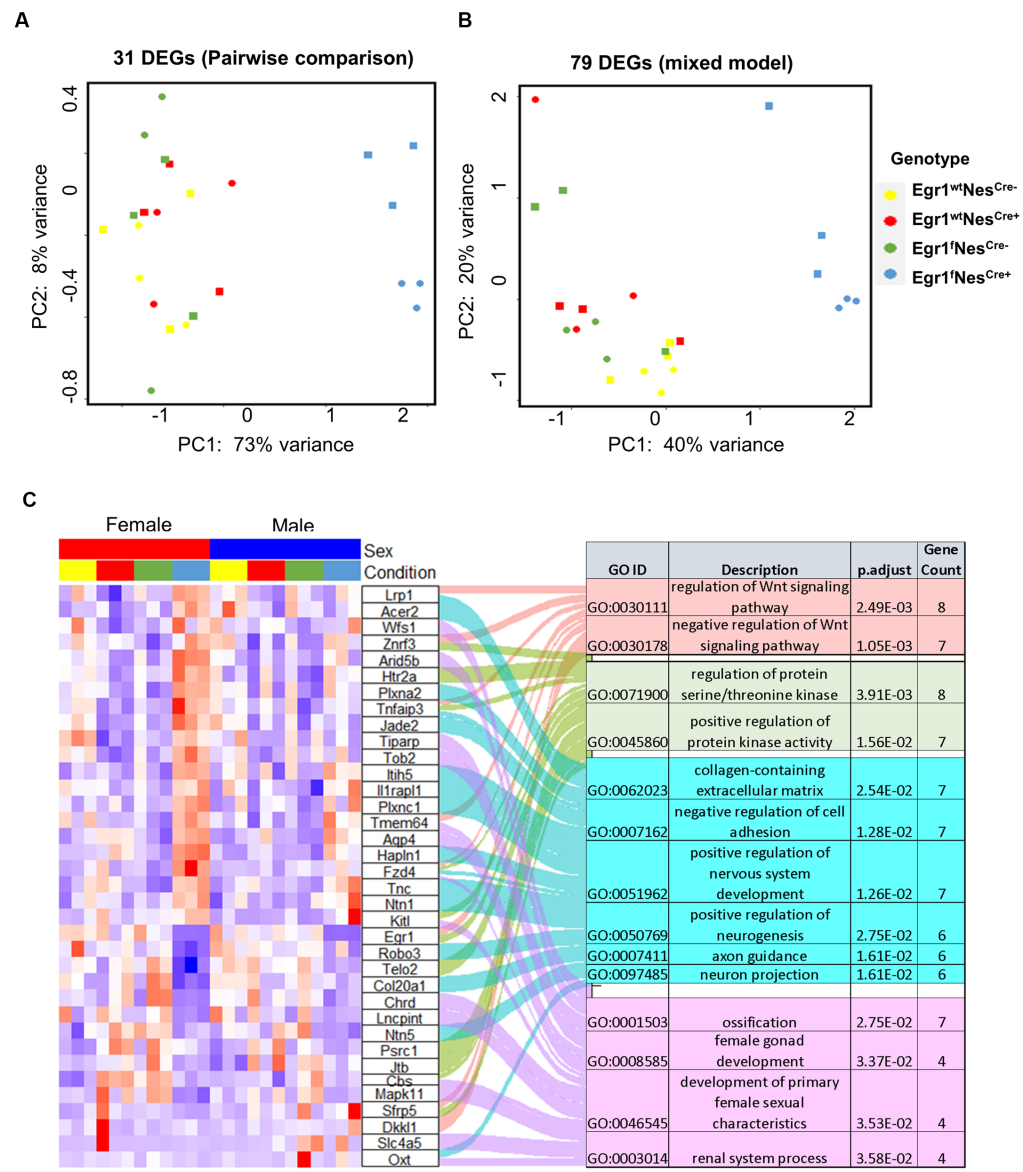
Differential expressed genes associated with Egr1cKO. **(A,B)** PCA analyses for 31 DEGs identified by pairwise comparison **(A)** and 79 DEGs identified by the mixed model **(B)**. **(C)** Heatmap of gene expression profiles for DEGs identified to be enriched in GO biological processed.

Lastly, we examined the influence of *Egr1* loss using mixed model. A total of 79 genes were identified with only six overlapped with 31 DEGs determined in pairwise comparison. The overlapped six genes in the two lists includes *Egr1*, Mitogen-activated protein kinases 11 (*Mapk11*), Contactin-associated protein like 5–2 (*Cntnap5b*), Iduronate 2-sulfatase (*Ids*), Tyrosine-protein phosphatase non-receptor type 14 (*Ptpn14*), and Gm42772. As one of the four p38 MAPKs, MAPK11 (p38β) plays an important role in response to extracellular stimuli. *Cntnap5b* gene encodes an extracellular matrix protein with function as a cell adhesion molecule and has been associated with dyslexia, a learning disability (Pagnamenta et al., 2010). PCA analyses were performed using the expression profiles of two gene lists, the 31 DEGs derived from pairwise comparison and the 79 genes derived from mixed model (Figures 6A,B). We found both gene sets were sufficient to separate the mice into two groups with or without *Egr1* loss. This result indicated that pairwise comparison and mixed model are useful for the identification of genes which might be associated with *Egr1* loss.

Further GO analysis for biological processes indicated that the union set of 104 DEGs linked to *Egr1* conditional knockout were enriched for Wnt signaling pathway, regulation of protein serine/threonine kinase activity, extracellular matrix components, axon guidance, and development of nervous system (Figure 6C). Interestingly, these biological processes are not independent from each other. Wnt signaling has been shown to be a critical pathway regulating neuronal extracellular matrix expression and axon terminal expansion (Wehner et al., 2017; Stanganello et al., 2019). On the other hand, the composition of extracellular matrix could dictate Wnt expression (du et al., 2016). Extracellular matrix molecules are key components in the formation of axonal tracts and both biological processes are involved in neuronal maturation and synaptogenesis (Barros et al., 2011). EGR1 is able to activate the Wnt/beta-catenin, promotes oncogenesis in tumoral cells (Zhang et al., 2021) but induces apoptosis in leukemia cells (Ma et al., 2020). Together with these findings, our results support that *Egr1* gene may regulate Wnt signaling in different types of cells to achieve distinct functions. Altogether, our study reported the indispensable role of *Egr1* in the regulation of Wnt signaling, extracellular matrix, and axon guidance in neuronal cell lineage. This provides an important mechanistic view on how *Egr1* contributes to synaptogenesis, neuron migration, and maturation.

## Discussion

The Nestin-Cre transgenic mice have been widely used to study gene functions in the central nervous system. However, previous studies report that Nestin-Cre driver mice have reduced growth rate and altered behavioral responses to stress (Declercq et al., 2015). Here we report that, a Nestin-Cre driver crossed to a floxed Egr1 mouse successfully abolished *Egr1* expression in hippocampal cells. Both *Egr1* loss and Nestin-Cre driver are associated with behavior abnormalities in transgenic mice. Previous studies found that complete Egr1KO mice have abnormal spatial memory and impaired contextual fear memory. Therefore, we performed a battery of behavior tests to monitor their responses to stress, learning, and memory. In this study we observed Egr1cKO driven by Nestin-Cre, pain responses and spatial novel object memory were not altered. Egr1cKO had some exploratory anxiety associated differences between sex, but these are difficult to interpret given the effects on activity in the open field. There was a clear behavioral difference between sexes and genotypes during the training and testing sessions for the contextual fear conditioning task. The males froze more during the context test than females, and the Egr1cKO group in both sexes froze more than controls. The Wnt signaling pathway in the hippocampus has been identified as a key driver of social avoidance in male mice following defeat stress (Wilkinson et al., 2011), along with other neuropathic disorders specifically in the hippocampus (Hussaini et al., 2014). Our study suggests that Egr1cKO could lead to abnormal Wnt signaling therefore causing behavioral changes that influence anxiety. Future study to define specific pathways linked with *Egr1* and Wnt signaling pathway should be conducted to further understand the links between molecular and behavior changes.

Our IHC and western blot results demonstrated that EGR1 was largely depleted from the Egr1cKO brain using the Nestin-Cre Loxp system. However, such a knockout is not hippocampal specific and brain regions other than hippocampus contribute to behavior responses. This limits our ability to interpret behavior changes observed with hippocampal gene expression analysis alone. In addition, *Egr1* is known to have a decreased expression in the brains of schizophrenia patients (Kimoto et al., 2014; Gallo et al., 2018). However, the blood samples from the sample patients with lower brain *Egr1* expression, showed an increase expression of *Egr1* (Kurian et al., 2011; Cattane et al., 2015). Such a phenomenon could be resulted from the attempt of our bodies to compensate for lower brain *Egr1* expression. Thus, other unknown compensatory mechanisms for the decreased expression of *Egr1* may exist and warrant further investigation.

To our knowledge, this is the first study systematically analyzing transcriptomes of Egr1cKO mice together with Cre, floxP, and wild type controls. Between the two sexes, only a handful genes were found to be differentially expressed in mouse hippocampus and the presence of Nestin-Cre or floxP loci alone did not introduce substantial changes. Notably, for 22 out of 24 mice used in this study, we have performed transcriptomic analysis for pituitary tissues as well. A total of 217 genes were identified to be differentially expressed between male and female pituitary tissues in wild type controls and such a number increased to approximately 2,000 in Egr1^f^Nes^Cre+^ mice (Swilley et al., 2023). Nestin-Cre or floxP alone showed significant influence on pituitary sex-linked gene expression patterns as well. Such a tissue-specific difference in sex-linked gene expression patterns observed in our studies raised an issue that some conclusions derived from transgenic mice may not be able to be generalized to both sexes and across tissues.

Despite using both pairwise comparison approach and a mixed effect model, we were only able to identify a small number of DEGs in hippocampus associated with distinct genotypes. In addition, a small fraction of overlapped DEGs was detected by both approaches indicated that the transcriptomic influence of *Egr1* loss in neuronal lineage cells is subtle at whole tissue level or covered by the transcripts derived from other types of cells present in hippocampus. Single cell RNAseq in future may help to provide a more complete picture. Regardless, for Egr1cKO, GO term analysis was able to identify several major biological processes enriched for the list of differentially expressed genes. Future studies would be desired to gain a better understanding of the functions of *Egr1* in distinct neuronal types when animals performing diverse memory tasks.

## Conclusion

Altogether, our results support that Nestin-Cre driver can efficiently remove *Egr1* genes in neuronal lineage cells in hippocampal cells. RNA-seq analysis revealed that Wnt signaling, and axon guidance pathways were disrupted by Egr1cKO. In addition, sex differences in behavior and gene expression were observed in the Egr1cKO mice and female mice are more sensitive to the loss of *Egr1* gene.

## Data availability statement

The datasets presented in this study can be found in online repositories. The names of the repository/repositories and accession number(s) can be found at: https://www.ncbi.nlm.nih.gov/geo/query/acc.cgi?acc=GSE232396.

## Ethics statement

The animal study was approved by Institutional Animal Care and Use Committee at Virginia Tech (Blacksburg, VA, USA). The study was conducted in accordance with the local legislation and institutional requirements.

## Author contributions

CS and HX conceived the experimental design. CS and ML were responsible for animal breeding and genotyping PCR. ML was responsible for the RNA isolation. CS was responsible for all behavioral experiments with guidance from GH and TJ, CS, and XX were responsible for immunohistochemistry. YZ and YL were responsible for mRNA-seq data processing and conducted the statistical evaluation. CS, GH, TJ, and HX interpreted the results and drafted the manuscript. All authors discussed the results, read, and edited the manuscript, and approved the final manuscript.

## Abbreviations

*Egr1*: Early growth response 1
*Egr1*cKO: Early growth response conditional knockout
LTD: Long Term Depression
LTP: Long Term Potentiation
EPM: Elevated Plus Maze
CFC: Contextual Fear Conditioning
PCA: Principal component analysis
GO: Gene Ontology
DEGs: Differential Expressed Genes
