# Supplementary figures and images for: Sex linked behavioral and hippocampal transcriptomic changes in mice with cell-type specific Egr1 loss
Source: Front Neurosci. 2023 Oct 19;17:1240209. doi: 10.3389/fnins.2023.1240209 (PMC10623684; doi:10.3389/fnins.2023.1240209)

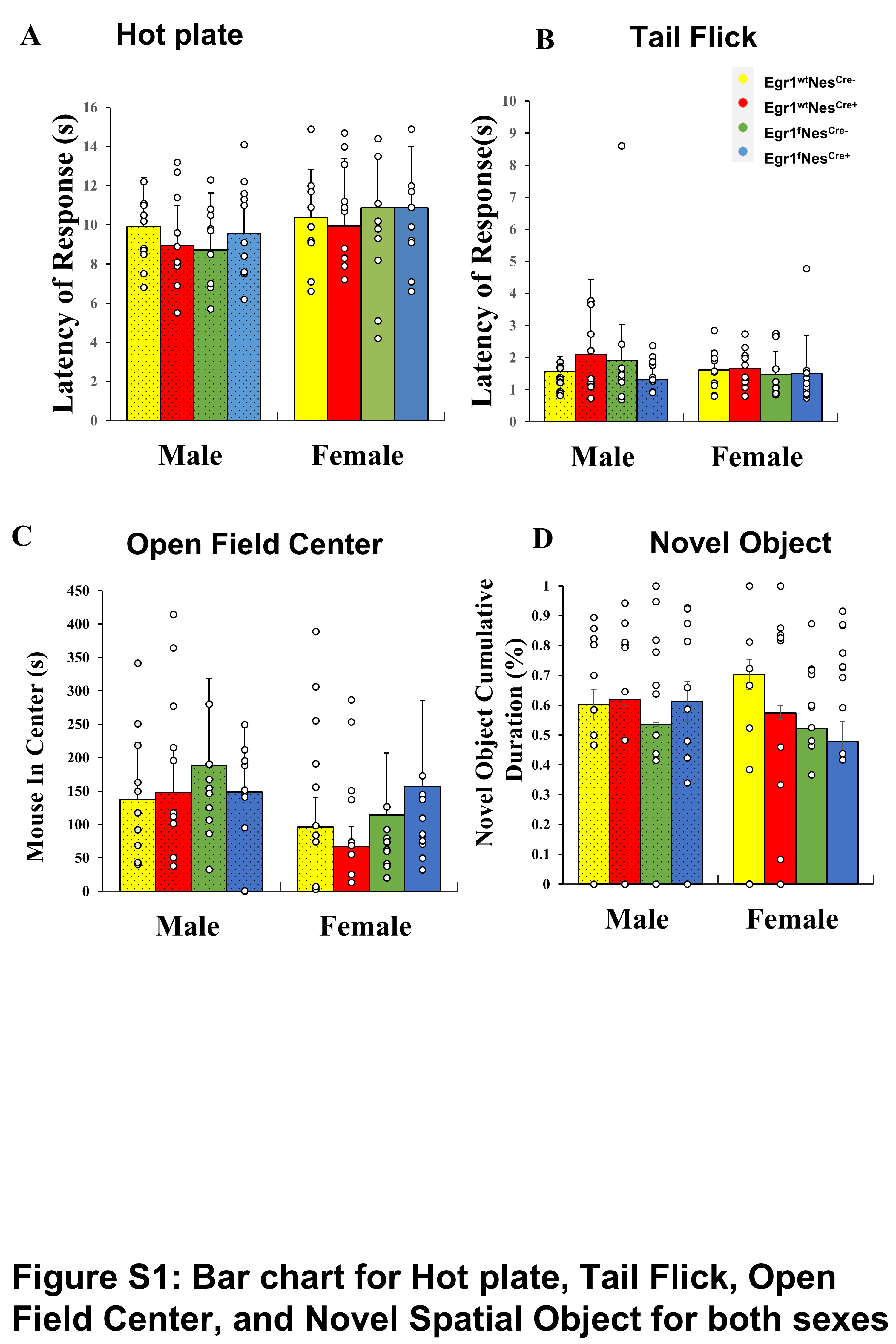

Supplement: Supplementary file 1 [file Image_1.tif]

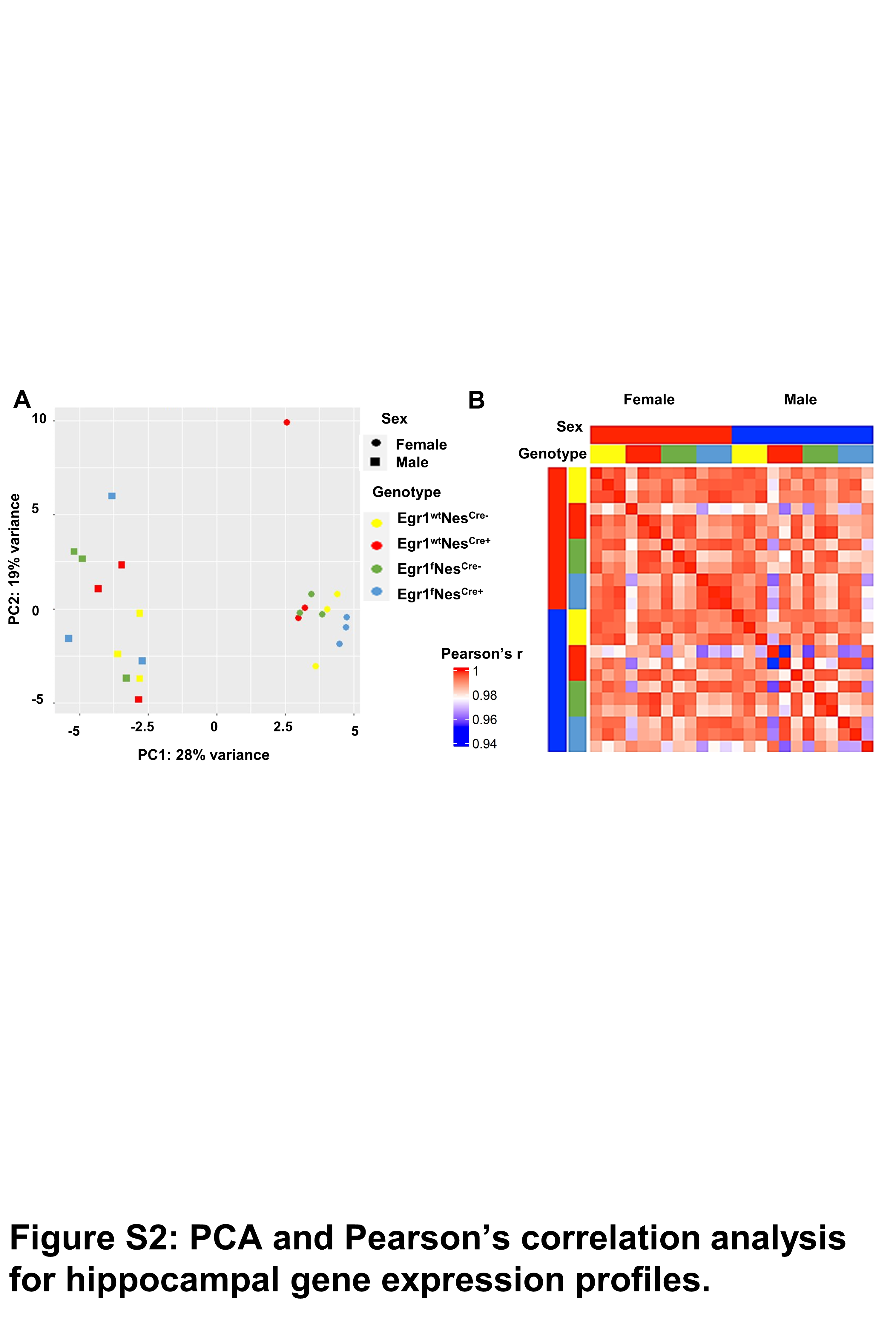

Supplement: Supplementary file 2 [file Image_2.tif]

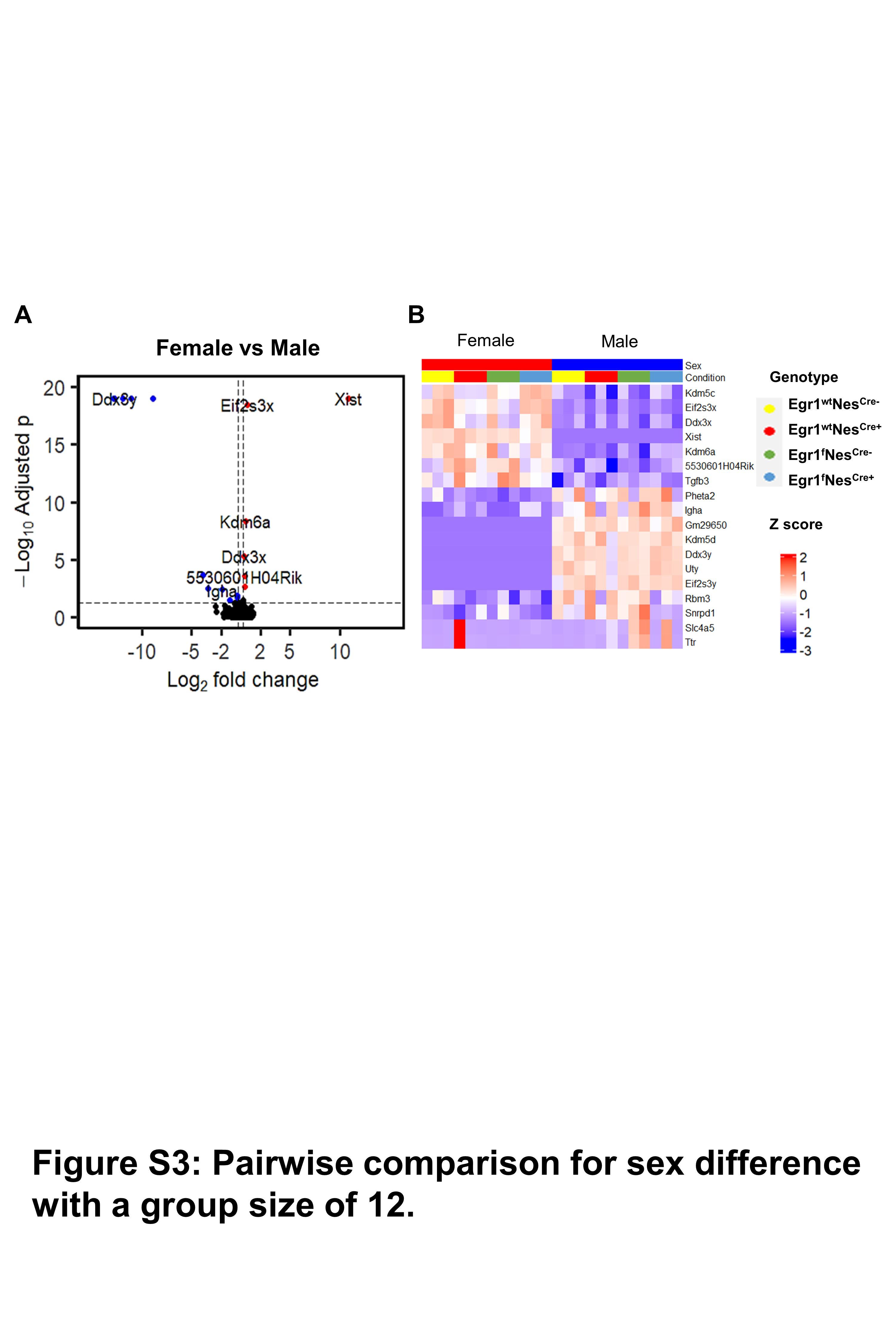

Supplement: Supplementary file 3 [file Image_3.tif]

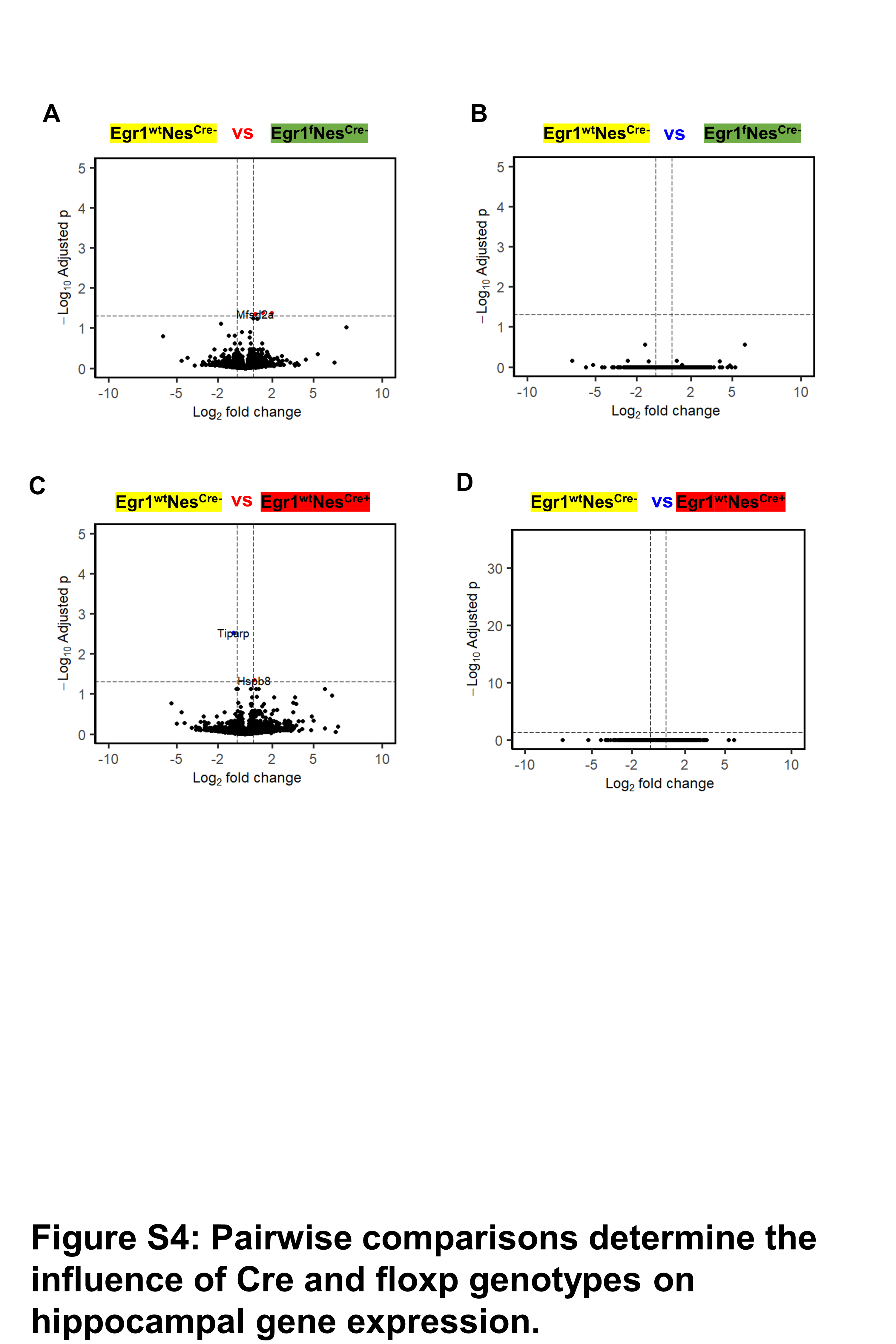

Supplement: Supplementary file 4 [file Image_4.tif]
